# MD simulation of the Tat/Cyclin T1/CDK9 complex revealing the hidden catalytic cavity within the CDK9 molecule upon Tat binding

**DOI:** 10.1371/journal.pone.0171727

**Published:** 2017-02-08

**Authors:** Kaori Asamitsu, Takatsugu Hirokawa, Takashi Okamoto

**Affiliations:** 1 Department of Molecular and Cellular Biology, Nagoya City University Graduate School of Medical Sciences, Nagoya, Aichi, Japan; 2 Molecular Profiling Research Center for Drug Discovery (molprof), National Institute of Advanced Industrial Science and Technology (AIST), Tokyo, Japan; 3 Division of Biomedical Science, Faculty of Medicine, University of Tsukuba, Tsukuba, Ibaraki, Japan; George Mason University, UNITED STATES

## Abstract

In this study, we applied molecular dynamics (MD) simulation to analyze the dynamic behavior of the Tat/CycT1/CDK9 tri-molecular complex and revealed the structural changes of P-TEFb upon Tat binding. We found that Tat could deliberately change the local flexibility of CycT1. Although the structural coordinates of the H1 and H2 helices did not substantially change, H1ʹ, H2ʹ, and H3ʹ exhibited significant changes *en masse*. Consequently, the CycT1 residues involved in Tat binding, namely Tat-recognition residues (TRRs), lost their flexibility with the addition of Tat to P-TEFb. In addition, we clarified the structural variation of CDK9 in complex with CycT1 in the presence or absence of Tat. Interestingly, Tat addition significantly reduced the structural variability of the T-loop, thus consolidating the structural integrity of P-TEFb. Finally, we deciphered the formation of the hidden catalytic cavity of CDK9 upon Tat binding. MD simulation revealed that the PITALRE signature sequence of CDK9 flips the inactive kinase cavity of CDK9 into the active form by connecting with Thr186, which is crucial for its activity, thus presumably recruiting the substrate peptide such as the C-terminal domain of RNA pol II. These findings provide vital information for the development of effective novel anti-HIV drugs with CDK9 catalytic activity as the target.

## Introduction

The number of individuals worldwide currently infected with human immunodeficiency virus type 1 (HIV-1) is estimated to be 35.3 million, and the number of deaths due to AIDS continues to rise in spite of effective antiviral agents [1]. Because the major targets of current anti-HIV-1 drugs include viral protease, reverse transcriptase, integrase, and viral attachment, none of which are the rate-determining step of viral replication, the emergence of drug-resistant viral clones due to the high rate of HIV-1 replication is inevitable [2]. Thus, the development of novel anti-HIV-1 therapeutics targeting viral transcription, which is essential for viral replication, remains a pressing need.

Transcription by the HIV-1 provirus is crucially regulated by the virus-encoded transcription factor Tat [3]. Tat requires a cellular transcription factor, positive transcription elongation factor b (P-TEFb), and the viral TAR RNA element formed at the 5ʹ-end of all HIV-1 mRNA transcripts [4,5,6]. It was revealed that P-TEFb contains a regulatory subunit, cyclin T1 (CycT1), and a catalytic subunit, cyclin-dependent kinase 9 (CDK9) [6,7,8], with CycT1 serving as the major interacting protein for Tat [9,10]. In other words, Tat recruits P-TEFb to the nascent viral transcripts, which allows CDK9 to commence the phosphorylation of the C-terminal domain (CTD) of RNA polymerase II (RNAPII), thus stimulating the transcriptional processivity of RNAPII and significantly augmenting viral transcription at the step of elongation [3]. In addition to P-TEFb, a number of transcriptional regulators, such as the superelongation complex [11,12,13,14], Hexim1 [15,16,17], 7SK snRNA [18,19], 7SK snRNP [14,20,21], and the basal transcription machinery [22,23], have been investigated in the context of Tat-mediated HIV transactivation. However, P-TEFb, among others, has long been the most extensively investigated molecular target of HIV transcription.

We and others have previously reported that the functional integrity of the TAR/Tat/P-TEFb complex is crucially involved in the activity of Tat [4,24,25,26]. We demonstrated that the N-terminal cyclin box, together with the Tat-recognition motif (TRM) of CycT1, is involved in Tat-mediated transcriptional activation by directly binding to CDK9 [24]. A number of studies on CycT1 mutants have revealed the distinct roles of various AA residues within CycT1 [25,26,27,28,29], and these findings support the functional integrity of the TAR/Tat/P-TEFb complex. Thus, Tat regulates P-TEFb activity and HIV transcription through a direct protein-protein interaction with CycT1.

In addition, a number of previous studies have demonstrated the regulation of CDK9 activity by a regulatory subunit that was later identified as CycT1 [30,31]. In the context of HIV transcription, Tat is responsible for this CycT1-mediated regulation of CDK9 activity by modulating the action of CycT1 [32]. CDK9 was originally identified as a Tat-associated kinase (TAK) or the cdc-2-like cyclin-dependent kinase PITALRE [33,34,35,36]. The activity of P-TEFb was shown to depend on the phosphorylation of several Ser and Thr residues on CDK9, particularly Thr186 and Ser175 located in the regulatory “T-loop” (amino acids 168–197). It is known that CDK9 T-loop phosphorylation is very low in resting CD4+ T-cells [37,38], further limiting P-TEFb activity. Upon T-cell activation, however, the T-loop is heavily phosphorylated, and its conformation changes, thus allowing entry of the substrate and ATP into the CDK9 catalytic pocket [39]. The phosphorylation of Thr186 in the CDK9 T-loop is involved in inducing its catalytic activity and binding to its negative regulator, 7SK snRNP [15,40]. Interestingly, protein phosphatase 1, which dephosphorylates Thr186, is reported to be involved in Tat-mediated transcription [41,42]. Thus, it is assumed that the reversible phosphorylation at Thr186 of CDK9 is critically involved in the regulation of P-TEFb activity [3]. In addition, the phosphorylation of Ser175 in the CDK9 T-loop also plays a regulatory role in altering the competitive binding of Tat to P-TEFb over BRD4 [43,44]. Moreover, the 3D crystal structure of the Tat/CycT1/CDK9 complex has recently been resolved, revealing the physical interaction between Tat and the CDK9 T-loop in addition to its interaction with CycT1 [45].

In order to study the structural background for the catalytic action of P-TEFb, a number of studies have revealed the molecular interaction of Tat and P-TEFb [4,24,25,27,28,46]. However, X-ray crystallography that has been utilized for these studied has generally limited ability in revealing the structural characteristics of protein complex and is considered to have limited chemical space. Since the Tat/P-TEFb complex in such studies utilized the CDK9 molecule with phosphorylated Thr186 that is considered to have regulatory role in its catalytic activity, it is suggested that the molecular flexibility in the vicinity of Thr186 is substantially limited because of the interaction with neighboring Arg residues (Arg 65 and Arg148) [45].

Thus, in this study we have attempted molecular dynamics (MD) simulation in order to search for the transient structure that is “hidden” in the crystallographic studies with the unphosphorylated Thr186 residue of CDK9. Interestingly, MD simulation revealed the dynamic action of proteins [47,48,49]. Toth et al. [49] observed the spontaneous opening and reclosing of the HIV-1 protease flaps based on the NMR structural data. In Filamia et al [50]., MD simulation of MAP kinase p38 was able to decipher the hidden intramolecular cavity that fits the effective inhibitor chemical. With regard to the application of MD simulation of protein-protein interaction (PPI), we have recently adopted molecular dynamics (MD) simulation in order to analyze the dynamic characteristics of the Tat-CycT1 protein-protein interaction (PPI) [26]. In addition, Jin et al. [51] also used MD simulation to reveal that HIV-1-Tat binding influences the phosphorylation of the CDK9 T-loop.

Here, we applied MD simulation to analyze the dynamic behavior of the Tat/CycT1/CDK9 tri-molecular complex and revealed the structural changes of P-TEFb upon Tat binding. The results indicate the possible role of Tat in modulating the structural changes of P-TEFb, in particular that of the “catalytic cavity” of CDK9.

## Materials and methods

### Molecular simulation

MD simulations were carried out using templates of the 3D structures of Tat/P-TEFb (PDBID: 3MI9) and P-TEFb, consisting of CycT1 and CDK9, which were extracted from the same crystal structure of Tat/P-TEFb (3MI9). In order to clarify the structural arrangement of the Tat/P-TEFb complex, the phosphorylated Thr186 in CDK9 within the 3MI9 model was computationally substituted with an unphosphorylated Thr. The Tat sequence in the PDB data (3MI9) was substituted to match the Tat sequence used in our laboratory [25,26,52]: the substituted residues include R7N, K29R, T23N, Q35Y, V36A, I39T, T40R, and A42G (the activities of these Tat molecules are considered nearly identical). Missing side chains, loops, tails, and mutations were built using the homology modeling module in MOE ver.20140901 (Chemical Computing Group Inc.).

The simulation was carried out using the Desmond ver. 3.8 package [53] with the OPLS2005 force field. SGI Rackalbe RP2 Standard-Depth Servers C2108-RP2 (Intel Xeon Processor E5-2670, 16CPU/node) at AIST were used as the computational hardware in this simulation. The initial model structure was refined using the protein preparation wizard in Maestro (Schrödinger, LLC) and placed into TIP3P water molecules solvated with 0.15 M NaCl. After minimization and relaxation of the model, the production molecular dynamics phase was performed for three independent 100-ns simulations in an isothermal-isobaric (NpT) ensemble at 300 K and 1 bar using Langevin dynamics. Long-range electrostatic interactions were computed using the Smooth Particle Mesh Ewald method. All system setups were performed using Maestro. Trajectory coordinates were recorded every 100 ps. The simulation trajectories were then analyzed for the sampled conformations in all structural models. The obtained trajectory was processed utilizing the AMBER11 tool ptraj for the calculations of RMSD and PMSF for protein RMSD. All molecular figures were generated using PyMOL (Schrödinger, LLC).

### Principal Component Analysis (PCA)

Principal component analysis (PCA) was performed on the 3D coordinates of Cα atoms of the TRRs [26] of CycT1 for MD trajectories of Tat/CycT1/CDK9 and CycT1/CDK9 complexes. The PCA results for the Tat/CycT1/CDK9 and CycT1/CDK9 complex structures were analyzed, and the conformational differences were visualized by plotting to the PC1 (primary component 1) and PC2 axes. PCA is a standard statistical method routinely used to identify variable correlations in a system from atomic fluctuations in an MD trajectory. The RMSD distance matrix of TRR Cα atoms for the combined trajectories of Tat/CycT1/CDK9 and CycT1/CDK9 structures was calculated after structure alignment of all Cα atoms to prepare the input matrix to PCA. PCA was performed using R (version 3.1.1.), which is a software language and environment for statistical computing and graphics.

### Concavity shape analysis of the protein structure

The likeliest active site pocket around the ATP-binding position and Thr186 within the representative structure from the MD simulations was identified using the SiteFinder module of MOE. The shape of the active site pocket was represented by small dummy atoms (α-spheres). The representative structures of Tat/P-TEFb and P-TEFb were each chosen from 3,000 trajectory coordinates (1,000 trajectories each for three independent run) by a k-means clustering algorithm (k = 1) using the AMBER11 tool ptraj command.

## Results

### MD simulations of the Tat/CycT1/CDK9 trimolecular and CycT1/CDK9 bimolecular complexes

**Fig 1:**
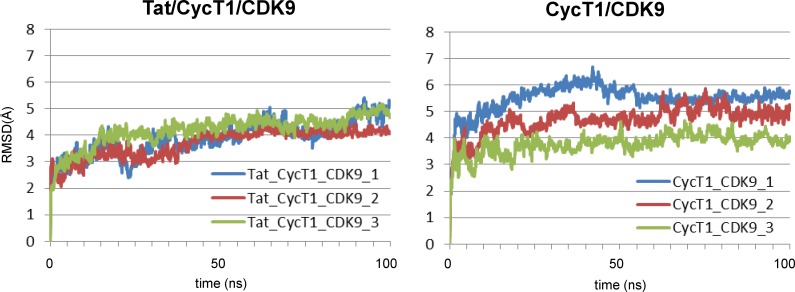
Molecular dynamics (MD) simulation. The time dependence of the root mean square deviations (RMSDs) of Cα atoms of Tat/CycT1/CDK9 (left) and CycT1/CDK9 (right) in the 100-ns MD simulations. For each complex, the results of three independent computations are shown (1, 2, and 3, for each complex).

The dynamic structural fluctuations of P-TEFb in the presence or the absence of Tat were analyzed by MD simulation for 100 ns. The structural fluctuation of the Tat/CycT1/CDK9 tri-molecular complex (3MI9) was analyzed using Desmond ver. 3.8 as described in Experimental Procedures for 100 ns (Fig 1, left panel). The fluctuation of the CycT1/CDK9 bi-molecular structure was similarly examined using the coordinates of 3MI9 without the Tat protein moiety as the starting structure (Fig 1, right panel). In both MD simulations, the plateau was reached after approximately 30–50 ns. Similar computations were repeated three times, and similar results were obtained within the limited variations. Although the addition of Tat did not significantly change the mean of the root mean square deviation (RMSD) value of Cα positions of Tat/CycT1/CDK9, it apparently reduced its variation, suggesting that Tat might restrict the structural fluctuation of CycT1/CDK9 complex.

### Comparison of Root Mean Square Fluctuations (RMSFs) of the CycT1 molecule within the tri- and bi-molecular complexes upon MD simulation

**Fig 2:**
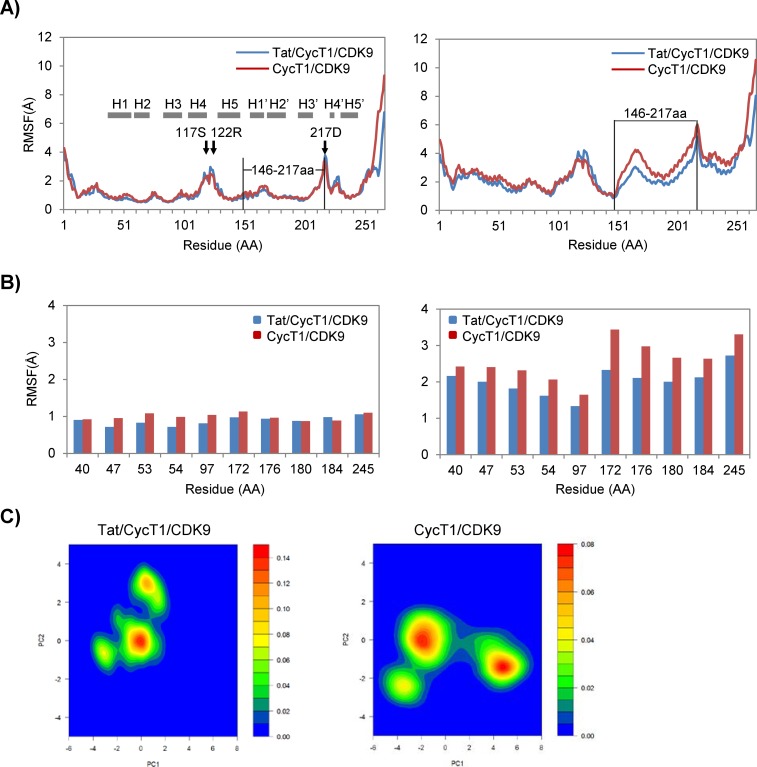
Tat restricts possible structural fluctuations of the CycT1/CDK9 complex. (A) RMSFs of CycT1 molecules (1–266 AA) during a 100-ns MD simulation of the Tat/CycT1/CDK9 (blue) and CycT1/CDK9 (red) complexes. Averages of three independent computations for each complex are shown. RMSF values of Cα atoms of CycT1 aligned to the initial coordinate of CycT1 are shown on the left; those using the initial coordinates of CDK9 for alignment are shown on the right. The locations of the α-helices are as follows (gray bars): H1, 31-52AA; H2, 54-69AA; H3, 80-64AA; H4, 101-102AA; H5, 125-143AA; H1ʹ, 153-163AA; H2ʹ, 168-184AA; H3ʹ, 193-207AA; H4ʹ, 221-224AA; and H5ʹ, 231-245AA. The positions of 117S, 122R, and 217D are indicated by black arrows. (B) Plots of RMSF values of CycT1 TRRs. RMSFs aligned with CycT1 (initial) and those aligned with CDK9 (initial) are shown on the left and the right, respectively. (C) Two-dimensional PCA projections of trajectories for the CycT1 TRRs obtained from the above MD simulations. The Cα coordinates for TRRs are plotted over PCA axes with principal component 1 and 2 (PC1 and PC2). The definition of the PCA axes was based on PCA calculations of all Cα coordinates with all CycT1 models, including Tat/CycT1/CDK9 and CycT1/CDK9. Using the RMSD distance data for only the TRR Cα atoms, each trajectory data set was plotted in accordance with PC1 and PC2 for the abscissa and ordinate, respectively. The 2D (PC1-PC2) plots of all 1,000 trajectories obtained from each triplicated MD simulation with Tat/CycT1/CDK9 (left) and CycT1/CDK9 (right) are shown. The probability distribution (in color) of the MD trajectories based on PCA was estimated using the kernel density estimation method in R version 3.1.1.

As shown in Fig 2A, we determined whether Tat could change the local flexibility of CycT1. As shown in the left panel, we found that the AA residues at 117S, 122R, and 217D were relatively flexible within the CycT1 molecule aligned with the original CycT1 (using the 3MI9 structure before MD simulation). The flexibility of these AAs did not significantly change in the presence or absence of Tat (compare the red and blue lines). Interestingly, the CycT1 region spanning from 146 to 217AA was continuously shifted when the CDK9 coordinate was used as the reference (Fig 2A, right panel). This structural shift was reduced when Tat was present, thus confirming the results of Fig 1. The H1ʹ-H2ʹ-H3ʹ region contained within the AA region spanning from 146 to 217 described above was located in the Tat-binding pocket that is formed together with the H1 and H2 helices. Although the structural coordinates of the H1 and H2 helices did not substantially change, those of H1ʹ, H2ʹ, and H3ʹ exhibited significant changes *en masse*. It was remarkable that the structural coordinate of the H3ʹ helix was also shifted although Tat directly interacts with the H1, H2, H1ʹ, and H2ʹ helices but not the H3ʹ helix. These findings also indicated the rotation of CycT1 relative to CDK9, which will be discussed later.

We previously identified the amino acid residues on CycT1, termed “Tat recognition residues (TRRs)” [26], involved in the interaction between Tat and CycT1. As shown in Fig 2B, RMSF values of those AA residues involved in Tat binding, or TRRs, were not significantly changed irrespective of the presence of Tat. However, when only the coordinates of these AA residues were statistically examined by PCA (Fig 2C), these coordinates of TRRs in the absence of Tat were classified into multiple distinct groups, suggesting the flexibility of the CycT1 structure before Tat-binding. Interestingly, the addition of Tat to P-TEFb dramatically reduced this flexibility, leaving one major group (Fig 2C, compare the left and the right panels).

### Stabilization of the CDK9 T-loop by Tat

**Fig 3:**
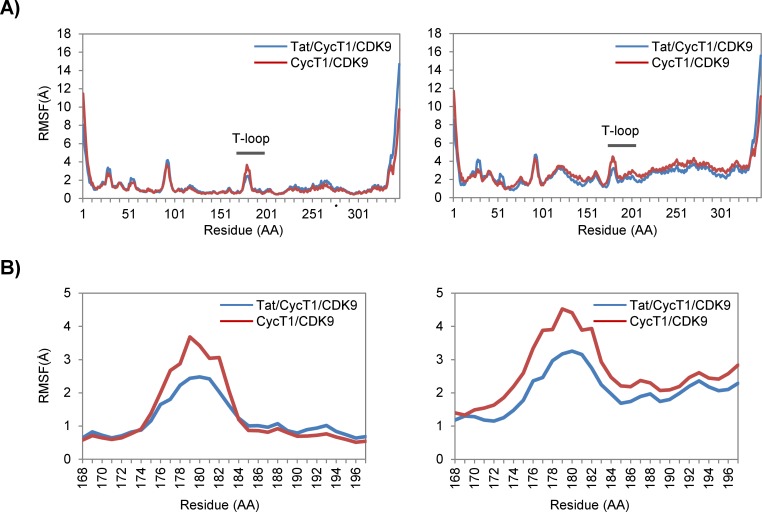
Tat restricts the possible structural fluctuations of the CDK9 T-loop. RMSFs of CDK9 molecules (1–345 AA) (A) and the CDK9 T-loop (168–197 AA) (B) during a 100-ns MD simulation of the Tat/CycT1/CDK9 (blue) and CycT1/CDK9 (red) complexes. Averages of three independent computations for each complex are shown. RMSFs of Cα atoms of CDK9 aligned with the initial coordinate of CDK9 are shown on the left. Those with the initial coordinates of CycT1 are shown on the right.

As shown in Fig 3, we examined the structural variation of CDK9 in complex with CycT1 in the presence or absence of Tat using the original CDK9 and CycT1 coordinates as the reference. Interestingly, Tat addition appeared to reduce the variability of the T-loop containing the Ser175 and Thr186 residues in CDK9 that are phosphorylated upon its activation (Fig 3, lower panels), although the other part of this molecule was not significantly affected. When the CycT1 structure was used as the structural reference, Tat binding similarly reduced the structural variability of the T-loop (Fig 3, right panel). It was assumed that this stabilization was due to the geometrical proximity of Tat and the CDK9 T-loop, which was considered crucial for the continuous phosphorylation of these residues.

### Comparison of the 3D structures of P-TEFb with or without Tat

**Fig 4:**
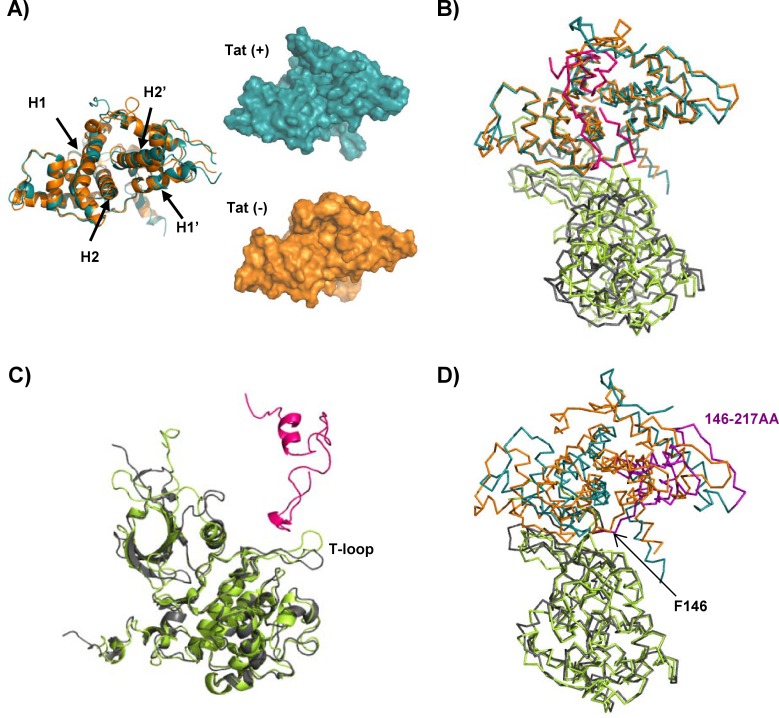
Comparison of the representative structures of the Tat/CycT1/CDK9 and CycT1/CDK9 models obtained by MD simulation. (A) Comparison of the depicted CycT1 structures observed for the Tat/CycT1/CDK9 (cyan) and CycT1/CDK9 (orange) complexes. Note that the cleft formed with the H1-H2 and H1ʹ-H2ʹ helices narrowed following Tat binding. Left, the superimposed CycT1 models; right, the surface representations of CycT1 molecules with (cyan) or without (orange) Tat. (B) Superimposition of CycT1. Tat/CycT1/CDK9: Tat (magenta), CycT1 (cyan), and CDK9 (dark green); CycT1/CDK9: CycT1 (orange) and CDK9 (light green). Note the rotation of CDK9 (see details in the text). (C) Superimposition of CDK9 showing the T-loop shift induced by Tat binding. (D) Superimposition of CDK9 showing the rotation of CycT1. Note that F146 of CycT1 stayed in the same position with or without Tat. The colors in C and D are the same as in B. The representative structures were each chosen from 3,000 trajectory coordinates (1,000 trajectories each for three independent runs) by the k-means clustering algorithm (k = 1) using the AMBER11 tool ptraj command.

The MD simulation results indicated the Tat-induced structural changes of P-TEFb. In Fig 4A and 4B, we superimposed the CycT1 structure in the presence (cyan) or absence (orange) of Tat. We found a narrowing of the Tat-binding cleft, formed between the H1-H2 and H1ʹ-H2ʹ-(H3ʹ) helices, upon Tat binding (compare the upper and bottom figures in Fig 4A). MD simulation revealed that this narrowing was induced by the internal shift of the H1ʹ and H2ʹ helices to the CycT1 core (compare the orange helices with the cyan helices in Fig 4A). We previously reported the internal movement of these helices upon MD simulation of only CycT1 molecule [26]. It was thus hypothesized that this internal movement might be crucial for Tat binding and the structural stabilization of the complex. When the CycT1/CDK9 complex was superimposed on CycT1, the CDK9 molecule appeared to rotate 5° (Fig 4B). This molecular rotation was previously reported by Tahirov et al. [45], thus corroborating our current MD simulation analyses.

As shown in Fig 4C and 4D, we analyzed the CDK9 structure in the presence (dark green) or absence (light green) of Tat. The T-loop appeared to shift away from Tat (Fig 4C). When the structure of the CycT1 molecule was assessed together (Fig 4D), only one AA, Phe146 of CycT1, stayed in the same position (also refer to Fig 2A, right panel). In contrast, the positions of AA residues between 147E and 217D were quite variable. The molecular rotation of CycT1 appeared to surround F146, which is located in the middle of 10 α-helices of CycT1, with the H1-H5 and H1ʹ-H5ʹ helices.

### Tat-induced stabilization of the CycT1/CDK9 complex

**Fig 5:**
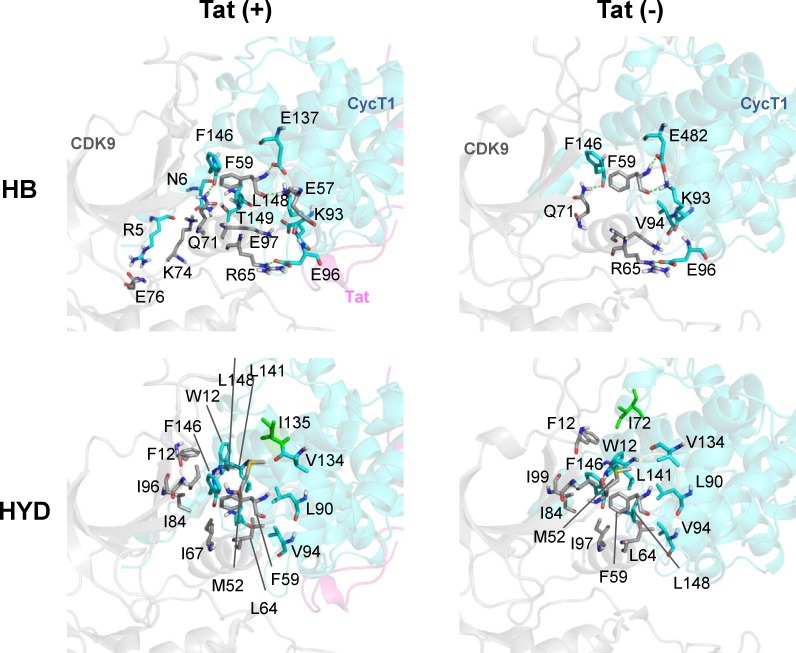
Atomic details of the principal interactions between CycT1 and CDK9 in the presence or absence of Tat. In order to identify the directly interacting residues, Contact Analyzer software (MOE ver. 20140910) was utilized. CDK9 is shown as a light gray ribbon with gray sticks, and CycT1 is shown as a light cyan ribbon with cyan sticks; the residues involved in hydrogen or hydrophobic bonds are depicted. Tat is shown as a light pink ribbon. The CycT1 residues affected by Tat binding through hydrophobic interactions are labeled in green. HB, hydrogen bond; HYD, hydrophobic interaction.

Fig 5 illustrates the atomic details of the principal interactions between CycT1 and CDK9 in the presence or absence of Tat. In contrast to the absence of Tat, Tat significantly increased the number of hydrogen bonds between these two molecules (from 5 to 11 hydrogen bonds; see the details in S1 Table). Thus, Tat stabilizes the CycT1/CDK9 complex by creating a significant number of novel hydrogen bonds. With regard to hydrophobic interactions in the CycT1/CDK9 complex, the addition of the Tat molecule appears to endow hydrophobic interactions in a more localized fashion. These findings indicate the molecular basis of Tat’s action to stabilize the CycT1/CDK9 complex. S1 Table summarizes the molecular details of the hydrogen bonding and hydrophobic interactions between CycT1 and CDK9 induced by Tat binding. Consistent with this finding from the MD simulation, we have previously reported that Tat induces increased binding affinity between CycT1 and CDK9 using image-based protein-protein interaction analysis in live cells [52].

### Induction of catalytic cavity within CDK9 by Tat alongside the PITALRE signature sequence

**Fig 6:**
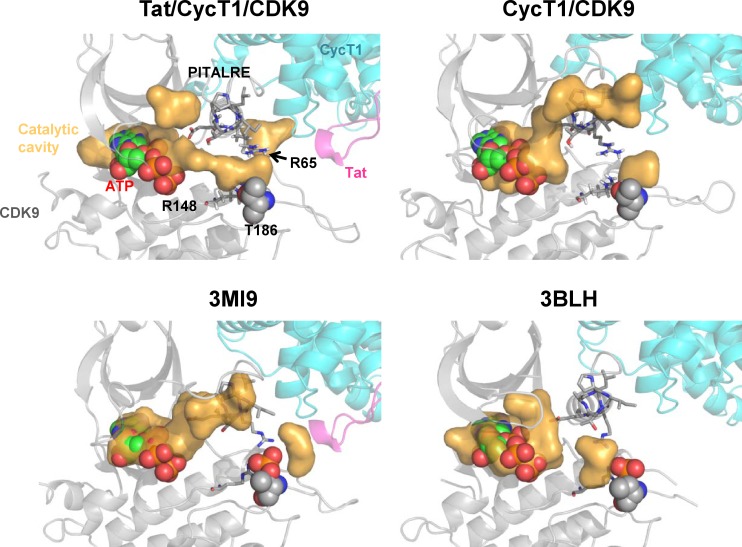
Induction of continuous catalytic cavity within the CDK9 molecule by Tat. CDK9, CycT1, and Tat are shown as gray, cyan, and pink ribbons, respectively. ATP is shown as a sphere with carbon atoms colored green, nitrogen atoms colored blue, oxygen atoms colored red, and phosphate atoms colored orange. The proposed ATP position was located in the binding site of CDK9 by structural alignment with the crystal structure of ATP-bound CDK9 (PDB ID: 3MIA). Thr186 is shown as spheres. The side chains of Arg85 and Arg148 in CDK9 are shown as sticks. The CDK9 catalytic cavity is shown in orange. Note the formation of a continuous catalytic cavity alongside the signature PITALRE sequence within the CDK9 molecule. The locations of cavities depicted in previous crystographic analyses (3MI9 and 3BLH, corresponding to the Tat/CycT1/CDK9 and CycT1/CDK9 complexes, respectively) are presented below.

The substrate specificity of CDK9 has been shown to be determined by the PITALRE sequence [33]. In order to analyze the effects of Tat, we applied MD simulation to analyze the molecular remodeling of the CycT1/CDK9 complex induced by Tat. As shown in Fig 6, Thr186 is located within the catalytic center of CDK9 [15,40,45] adjacent to PITALRE and the catalytic cavity (drawn in yellow) whose structure is not continuous with Thr186 (Fig 5, right panel). The addition of Tat appears to induce the interconnection of two internal Arg residues of CDK9, Arg65 and Arg148, with Thr186, thus forming a continuous catalytic cavity that links Thr186 and the substrate ATP molecule. It appears that the PITALRE signature sequence flips the vacant kinase cavity (left panel) to become connected with Thr186 (right panel), thus presumably recruiting the substrate peptide such as the C-terminal domain of RNA pol II (not shown in the figure). The liberated pyrophosphate (PPi; red) moiety from ATP might move through the continuous catalytic cavity to target the substrate.

## Discussion

It has been proposed that HIV-specific transcriptional regulation could be an ideal target for an effective AIDS therapy, as it would block the rate-determining step of viral replication and prevent the generation of drug-resistant viral clones [54]. In this study, we analyzed the dynamic molecular behavior of Tat during the activation of the host transcription elongation factor P-TEFb by applying MD simulation to clarify the dynamic structural changes of CycT1 and CDK9 upon binding to Tat. We observed that Tat binds to the CycT1 cleft that is formed by two sets of α-helices, namely the H1-H2 and H1ʹ-H2ʹ-H3ʹ helices, thus confirming the initial finding by Tahirov et al. [45]. Interestingly, we also found that upon Tat binding, this cleft is narrowed by primarily shifting the H1ʹ-H2ʹ-H3ʹ helices. More importantly, our MD simulation reveals, for the first time, that the accumulation of these subtle structural changes culminated in the formation of a continuous catalytic cavity that links the kinase substrate ATP to the CDK9 Thr186 that is located at its catalytic center alongside the signature sequence PITALRE [15,39].

In the present study, we found that the CycT1 H1ʹ-H2ʹ-H3ʹ helices lost flexibility in the presence of Tat, thus stabilizing the Tat/CycT1 complex. In the absence of Tat, the H1ʹ-H2ʹ-H3ʹ helices relatively opened up, with Phe146 as the symmetric center of this movement. We have previously reported that the flexibility of the H1ʹ and H2ʹ helices of CycT1 was crucial for the Tat-mediated transcriptional activation of HIV-1 in a study in which we analyzed the effects of the substitution of crucial AA residues [26]. We also proposed a pre-existing model where the putative Tat-binding pocket is transcendentally present on the molecular surface of CycT1, which was confirmed by the present MD simulation [26,55]. As shown in Fig 2C, Tat appears to choose one principal structure conformed by TRRs, whereas there are three possible structures for the TRRs in the absence of Tat.

More importantly, we observed the loss of flexibility of the CDK9 T-loop in the presence of Tat (Fig 3). Tat appears to restrict the flexible movement of the T-loop structure (Fig 4C). This leads to the formation of a continuous catalytic cavity that could liberate PPi from ATP to attack Thr186 through two Arg residues (Arg65 and Arg148) and then eventually the target C-terminal domain (CTD) of RNA polymerase II. It remains to be clarified whether the CTD moiety could actually be brought close to the Tat/CycT1/CDK9 complex upon co-crystallization. It is expected that the CDK9 catalytic center could be located in close proximity to the Ser2 residue within the CTD heptad sequence. Furthermore, the role of the signature sequence PITALRE could be to act as a fulcrum for the trans-generation of the catalytic cavity.

Our current MD simulation revealed a +5° rotation of CDK9, which was consistent with a previous report by Tahirov et al. [45], although they could not demonstrate the continuous structural extension of the catalytic cavity. This suggests the transient formation of such a cavity upon the dynamic movement of the CycT1/CDK9 complex in the presence of Tat. It is noted that this structural change of P-TEFb might be rather specific for Tat because other transcriptional activators such as AFF4 do not involve CDK9 kinase activity [56].

These findings should provide the structural basis for the development of a Tat-specific inhibitor that can not only inhibit the explosive replication capability of HIV but also prevent the rapid emergence of viral clones resistant to conventional anti-HIV regimens. Further studies are needed to clarify the details of the target structure against which novel anti-Tat compounds should be developed.

## Supporting information

S1 Table Inter and intra-helical hydrogen bonds within Tat/CycT1/CDK9 and CycT1/CDK9 representative structures. (DOCX)

## Abbreviations

CDK9: cyclin dependent kinase 9
CycT1: Cyclin T1
HIV-1: human immunodeficiency virus type 1
MD: molecular dynamics
PCA: principal component analysis
P-TEFb: positive transcription elongation factor b
PPI: protein-protein interaction
RMSD: root mean square deviations
RMSF: root mean square fluctuations
TRM: Tat-recognition motif
TRRs: Tat-recognition residues
