# Supplementary material for: MD simulation of the Tat/Cyclin T1/CDK9 complex revealing the hidden catalytic cavity within the CDK9 molecule upon Tat binding
Source: PLoS One. 2017 Feb 8;12(2):e0171727. doi: 10.1371/journal.pone.0171727 (PMC5298246; doi:10.1371/journal.pone.0171727)
Supplement: S1 Table — (DOCX) [file pone.0171727.s001.docx]

| Tat/CycT1/CDK9 | |  |  |  |  |
| --- | --- | --- | --- | --- | --- |
|  | Type | Chain | Residue | Chain | Residue |
| 1 | HB | CDK9 | E57, O | CycT1 | K93, NZ |
| 2 | HB | CDK9 | F59, O | CycT1 | K93, NZ |
| 3 | HB | CDK9 | F59, N | CycT1 | E137, OE1 |
| 4 | HB | CDK9 | R65, NE | CycT1 | E96, OE1 |
| 5 | HB | CDK9 | K68, NZ | CycT1 | E95, OE1 |
| 6 | HB | CDK9 | Q71, NE2 | CycT1 | F146, O |
| 7 | HB | CDK9 | Q71, OE1 | CycT1 | L148, N |
| 8 | HB | CDK9 | Q71, OE1 | CycT1 | T149, OG1 |
| 9 | HB | CDK9 | K74, NZ | CycT1 | R5, O |
| 10 | HB | CDK9 | K74, NZ | CycT1 | N6, OD1 |
| 11 | HB | CDK9 | E76, OE1 | CycT1 | R5, NH1 |
| 12 | HYD | CDK9 | F12, CD2 | CycT1 | W12, CZ2 |
| 13 | HYD | CDK9 | M52, CE | CycT1 | V134, CG2 |
| 14 | HYD | CDK9 | M52, CE | CycT1 | I135, CG1 |
| 15 | HYD | CDK9 | F59, CD2 | CycT1 | L141, CD1 |
| 16 | HYD | CDK9 | F59, CZ | CycT1 | F146, CE1 |
| 17 | HYD | CDK9 | L64, CD2 | CycT1 | L90, CD1 |
| 18 | HYD | CDK9 | L64, CD2 | CycT1 | V94, CG2 |
| 19 | HYD | CDK9 | L64, CD2 | CycT1 | L141, CD1 |
| 20 | HYD | CDK9 | L64, CD2 | CycT1 | L148, CD1 |
| 21 | HYD | CDK9 | I67, CD1 | CycT1 | F146, CD1 |
| 22 | HYD | CDK9 | I67, CG2 | CycT1 | L148, CD1 |
| 23 | HYD | CDK9 | I84, CD1 | CycT1 | F146, CB |
| 24 | HYD | CDK9 | I99, CG1 | CycT1 | F146, CZ |

HB, hydrogen bond; HYD, hydrophobic interaction.

| CycT1/CDK9 |  |  |  |  |  |
| --- | --- | --- | --- | --- | --- |
|  | Type | Chain | Residue | Chain | Residue |
| 1 | HB | CDK9 | M1, N | CycT1 | Q128, OE1 |
| 2 | HB | CDK9 | Q4, NE2 | CycT1 | D132, OD2 |
| 3 | HB | CDK9 | Y5, OH | CycT1 | D132, OD2 |
| 4 | HB | CDK9 | D6, N | CycT1 | Q77, OE1 |
| 5 | HB | CDK9 | S7, N | CycT1 | Q77, OE1 |
| 6 | HB | CDK9 | E9, O | CycT1 | Q73, NE2 |
| 7 | HB | CDK9 | F59, O | CycT1 | K93, NZ |
| 8 | HB | CDK9 | F59, N | CycT1 | E137, OE1 |
| 9 | HB | CDK9 | R65, NH2 | CycT1 | E96, OE1 |
| 10 | HB | CDK9 | K68, NZ | CycT1 | V94, O |
| 11 | HB | CDK9 | Q71, NE2 | CycT1 | F146, O |
| 12 | HYD | CDK9 | V8, CG2 | CycT1 | F78, CZ |
| 13 | HYD | CDK9 | V8, CG1 | CycT1 | I139, CD1 |
| 14 | HYD | CDK9 | F12, CD2 | CycT1 | W12, CH2 |
| 15 | HYD | CDK9 | F12, CE2 | CycT1 | I72, CD1 |
| 16 | HYD | CDK9 | M52, CE | CycT1 | V134, CG1 |
| 17 | HYD | CDK9 | F59, CD1 | CycT1 | L141, CD1 |
| 18 | HYD | CDK9 | F59, CE1 | CycT1 | F146, CE1 |
| 19 | HYD | CDK9 | L64, CD2 | CycT1 | L90, CD1 |
| 20 | HYD | CDK9 | L64, CD2 | CycT1 | V94, CG2 |
| 21 | HYD | CDK9 | L64, CD2 | CycT1 | L148, CD1 |
| 22 | HYD | CDK9 | I67, CD1 | CycT1 | F146, CB |
| 23 | HYD | CDK9 | I67, CG2 | CycT1 | L148, CD1 |
| 24 | HYD | CDK9 | I84, CG2 | CycT1 | F146, CD2 |
| 25 | HYD | CDK9 | I99, CG2 | CycT1 | F146, CZ |

HB, hydrogen bond; HYD, hydrophobic interaction.

The assignments of atoms involved in hydrogen bonds (HB) in No. 1 to 9 of this table were excluded from the table because these atoms, which are in residues Met1 to Glu9, are located in the N-terminus of CDK9.
